# Central Dental Association of Northern New Jersey

**Published:** 1888-03

**Authors:** 


### CENTRAL DENTAL ASSOCIATION OF NORTHERN NEW JERSEY.

---

REPORTED FOR THE INDEPENDENT PRACTITIONER.

Dr. F. T. Van Woert, of Brooklyn, read a paper on Crown and Bridge-Work. (See page 121.)

Dr. Van Woert made the following additional remarks during and after the reading of his paper:

Since adopting this system of crowning, I find that a similar crown had been devised by Dr. Richmond. It is an all porcelain crown; the labial and palatine surfaces of the root are beveled off, and the crown is supposed to fit that bevel. The principle is, I think, virtually the same, although I knew nothing about this when I devised mine, and I am sure Dr. Richmond knew nothing of my system.

I do not see how it is possible to grind such a crown to the root. After you have the root prepared, you can prepare the crown to make a true union between it and the root. Dr. Richmond may be very successful with his crown, and I have no doubt he is, but I do not think it is a practical thing in the hands of the general practitioner. It is beyond my skill, and I want something which I can manipulate with a fair chance of success.

Concerning bridge-work, so long as there is a question of the validity of the bridge patents I do not want to have much to do with it. But with a crown of this kind, the two upper canine roots would support a bridge as well as if they were banded. With that overhanging edge, it would be impossible to break it, and where it is practical at all, I think it is much easier to make than a band crown.

*Dr. Stockton*—Is it not rather expensive to fill bicuspid crowns up with gold?

*Dr. Van Woert*—You generally have plenty of scrap gold in the office that you can use for that purpose. I have here a complete copy of a chapter in Fitch's Dental Surgery, which describes this bridge work, and at some future day Dr. Parker will be only too glad to show you the book containing it. I do not want you to infer that I claim this to be a better crown than Dr. Knapp's. I confess that I am not the mechanic that Dr. Knapp is, and therefore I have to make something that I can use, and that will be equally serviceable.

*Dr. Osmun*—I have examined Dr. Van Woert's work carefully, and I think it has some marked advantages over the band method. I have found that these band crowns, where the band goes under the gum any distance, are a source of irritation, and I have gone back to my first love, the Bonwill and the Howe crowns. In this all gold crown I must say that there is a great waste of gold, but Dr. Van Woert was always a very generous fellow, and he throws in the gold very freely. I think that the shell crown, stiffened with eighteen carat gold solder on the inside and then put on in the usual manner, will meet all the requirements that a solid gold crown will, and at a great deal less expense.

*Dr. Evans*—I have a work of Dr. Fitch that describes the identical things which the gentleman has quoted. I showed it at a meeting of the First District Society in New York, two years ago. But the bridge-work done now is entirely different from that which is explained in that work. It is useless to say it is the same thing.

I am not an advocate of patents. I have patented some things myself, but I have extended their use to the profession. I refer to the contour seamless crown of my invention. Dr. Low supports a bridge on two points to which it is attached with cement, and to that bridge which spans the space artificial teeth are fixed in a rigid manner. Nothing like this was ever done before he applied for his patent. I have seen what you would call bridge-work dating as far back as 1867—such a piece as Dr. Fitch describes. But it has no relation to the bridge-work of the present day, and we dentists will never win a suit against the Tooth Crown Company on any such argument as that. The courts will not accept it. You must keep down to what the patent really is.

As to the crown that has been presented here, I have made crowns on that principle for years. I trim the root as he describes, take an impression of the end of the root with some moldine in a tube which is curved so as to fit over it, and from this I make a die. I then hammer over this a piece of lead, which gives the exact shape of the root. I then trim away the labial portion, if it laps over much. When fitted and put in place it makes a most excellent crown. It is in principle about the same thing as that shown here.

*Dr. Van Woert*—I think the gentleman misunderstood me. In speaking of that book I distinctly said that several teeth were supported on a plate by means of pivots or screws in the stumps of two or more teeth. If this is not a bridge I do not know what is. It is supporting several teeth across a broken arch without touching the gums. With respect to the making of crowns, every man has his own method. As for myself, to take an impression of the root and work from that impression only complicates matters, and I cannot do as well as I can to work from the root direct. The gentleman who has just spoken may be able to work from an impression with accuracy, but I cannot. I simply give you my method, which, to me, is an easy and good one.

*Dr. Evans.*—I first take an impression, then stamp a cap and adjust this to the root. That facilitates the operation considerably. It fits at all points. Regarding bridge-work, I do not mean to say that the principle of the bridge is new. On the contrary, it is one of the first principles that ever was adopted in the insertion of artificial teeth. You will find it mentioned away back in Roman and Grecian history. But the improvements upon that principle, as embodied in the patents of the International Tooth Crown Company, are new. That is the real point in this matter. I have a copy of a book by Dr. Fitch which gives such a description of a bridge as has been read by Dr. Van Woert, but my copy was published in New York, not in Philadelphia. I think Dr. Bödecker has a work by the same author.*

---

*The work of Dr. Fitch is somewhat rare, but it is not unique. We have a copy, and there are doubtless others in existence that are not mentioned. The first edition was published in New York, in 1829, the second in Philadelphia, in 1835.—Editor.

*Dr. Weld.*—I would like to ask the doctor if the crown he advocates preserves the root as well as a crown which is associated with a band; whether a band in connection with the cement is not an additional security to the root of the tooth.

*Dr. Van Woert.*—If it were properly trimmed below the margin of the gum, it might be better. If you get a perfect union between the root and the crown, I think it will be successful in ninety-nine cases out of a hundred, provided the root is sound. Dr. Stockton seems to think that a shell crown is as good as a solid gold crown. Possibly it is, but to make a die and strike up a crown takes more time than it does to burnish in the platinum and fill with gold, and the time is worth a great deal more than the gold. That is the reason I adopted this system—simply to save time. I can make a crown of that kind and put it on in three-quarters of an hour.

*Dr. Osmun.*—I have been wondering if a bridge made on this principle, with the cap cemented to the two attachments, would be covered by the patents of the International Tooth Crown Company. The Low patent covers a band around a tooth, cemented with some kind of cement.* A cap over a root, with a bridge thrown from it to another of the same kind certainly is not a band around a tooth. The important question is, whether under the Low patent the International Tooth Crown Company can compel dentists using this cap to take a license and pay royalties. I am not a believer in bridge-work in any sense of the word. I have put in a few bridges and have paid the royalties on them, and in every case, with a single exception, the roots have loosened in their sockets. The crowns have not loosened from the roots. The one that I relied upon as the best piece came in the other day, and the roots were so loose that I shall have to extract them. The question is, whether this device that has been presented by Dr. Van Woert would not obviate the necessity of paying royalties under the Low patent, for those who like to do bridge-work. Personally I do not

* The vital paragraph of the Low patent reads as follows:

"What I now claim as new is: The herein described method of inserting and supporting artificial teeth, which consists in attaching said artificial teeth to continuous bands fitted and cemented to the adjoining permanent teeth, whereby said artificial teeth are supported by said permanent teeth without dependence upon the gum beneath."—Editor.

believe in making two or three broken down roots do the work of seven or eight teeth.

*Dr. Luckey.*—I am not quite clear as to how Dr. Van Woert makes the union between the crown and the root, and where the cement goes. In fixing the metal cap over the end of the root, do you make allowance for the cement between them?

*Dr. Van Woert.*—It is but a very thin film and would take years to wash out. I have yet to see a root that is decayed under such a cap, except through defective workmanship. I have had them decay, but it was my own fault and not that of the crown. If the gold cap be properly adjusted to the end of the root, and fits accurately, I doubt very much whether caries will ever be found there.

*Dr. Luckey.*—You depend for your union upon the little cement that finds its way into the joint ?

*Dr. Van Woert.*—Yes.

*Dr. Luckey.*—Would you unite a Logan crown in that way ?

*Dr. Van Woert.*—I think you would get the same result, provided the union was as nearly perfect.

*Dr. Palmer.*—Speaking of the gold crown makes me think of a crown that was described in the October number of the *Cosmos*, in the construction of which the Logan crown is used, combined with a cap, forming a perfect all-porcelain crown. The gentleman describing that crown cuts the root off straight across, a little below the margin of the gum, then makes his cap for it, similar to but very much shorter than the Richmond crown, and with the same diameter at this point as in the centre. After the necessary curving of the band beneath the gum and fitting it to the root, it is driven into position. He bevels the roots off the same as in setting the Bonwill crown, using very thin gold and working it down with burnishers. He gets a cap smaller in diameter, and fitting very accurately to the end of the root, then punches a hole through it, and in this canal fits a pin; then taking a Logan crown he cuts the pin off just about even with the edges of the depression in the crown, and flows on pure gold. That he fits perfectly to the outline of the cap or root, then cuts the end or heel of the Logan crown off so that there is just the least opening at the back, fastens the two together with wax and invests them. There is a little piece of platinum carried in there as a guide or leader, and a small piece of solder unites the pure gold to the cap, and if it has been crowned so that the outside fits snugly to the cap, it is a very nice piece of work.

*Dr. Meeker*—In making a crown tooth the essential points are strength, ease and facility in doing the work. We do not want any more exertion than is necessary, for we grow old fast enough at the best. I think the principles of strength of the work and ease in doing it are fully illustrated in the Logan and Bonwill crowns, more especially the Logan crown.

Another point is the preservation of the root, because you have nothing going under the margin of the gum, and if we grind away the root, when the crown is driven to place the cement will ooze through. I have made gold and porcelain crowns, and spent much time on them, but I find I can make eight or ten dollars easier and quicker by using this crown, and it gives just as good satisfaction.

*Dr. Stockton*—I am very much interested in this presentation to-night, and I hope very much from it. At our last meeting Dr. Genese, of Baltimore, spoke about and presented pivot teeth constructed on a principle just the reverse of this, having a countersink instead of a bevel, and they have been in use for ten years at least. It has been very satisfactory, but I think this an easier and better way of putting a pivot tooth on. The objectionable point, as has been stated here to-night, is that in nine cases out of ten, the band does not fit the root. Nothing of that kind can occur in a tooth made like this one ; everything is free and clear, almost like a natural tooth, and I think they are certainly preferable. In answer to Dr. Osmun's question, I certainly think, from a layman's point of view, that this method of attachment would not infringe the bridge patent, because that distinctly describes a band around the root.

*Dr. Osmun*—I have noticed that with banded crowns there is always more or less inflammation around the gums after a time. I have seen them from the hands of men who are splendid workmen, the mechanical part being beautifully done, but in almost every case there has been more or less inflammation around the gum.

*Dr. Stockton*—I do not know just how many years it is since a number of you came to my house to see Dr. Richmond put on three crowns. On Saturday last the gentleman for whom these crowns were inserted was in my office. I have reset the right central; the left central is just as Dr. Richmond put it on, but the gum has receded so that the root shows all around the band. It looks very badly indeed, and the crown will have to be replaced. I think that very often there is recession of the gum when bands are used.

*Dr Baldwin*—Would not that occur in any tooth that had been crowned, or in a great many cases of pulpless teeth?

*Dr. Stockton*—I think the band impinges upon the peridental membrane and drives it up.

*Dr. Meeker*—If you destroy the peridental membrane I do not see how the gum can be prevented from receding.

*Dr. Baldwin*—The points to be looked to in crown work are, to get the work as true to nature as possible, to do it as easily as possible, and have it as firm and serviceable as possible. I believe an all-porcelain crown is the most true to nature of any, and I think you will agree with me that a banded crown is the firmest and best. I think you will also admit that the striking up of a crown in one piece is the easiest and best way to make a crown. In that case no soldering is necessary. That method of crowning, as you will recollect, has been described in the American system of dentistry, as Dr. Evans does it, only he applies it to another tooth.

One important point in this connection has been overlooked, and that is that in porcelain and gold crowns the secretions of the mouth penetrate between the porcelain and the gold, and after a time it becomes offensive. In the union of the Logan crown, the phosphate of zinc being above as well as below, the action of the secretions of the mouth is prevented. If we can fit a crown to a root so that there will be a film of phosphate between them to preserve the root, we surely can fit a band in the same manner, and if it should not be accurately fitted, that little film of phosphate that does the work in the other case will certainly serve for the band.

*Dr. Luckey*—My experience has been that the easiest to make and the strongest crown is the one inserted on the same principle that has been described in the paper, trimming away the root and hollowing out the crown so that the cement is thickest and firmest at that point, the root being cut out so that it makes a cup shape.

On motion, the subject was passed.

